# Humulus Lupulus in Intermittent Fever

**Published:** 1870-10

**Authors:** 


Humulus Lupulus in Intermittent Fever.—Dr. W. Y. Godberry, of Benton, Miss. (*The Jour. Materia Medica*), regards the anti-periodic power of humulus lupulus as equal to that of any other article in the Materia Medica, except quinia, and has often found it to succeed in arresting intermittents after quinia had failed.—*Medical Record.*

